# LncRNA-mRNA co-expression analysis discovered the diagnostic and prognostic biomarkers and potential therapeutic agents for myocardial infarction

**DOI:** 10.18632/aging.202713

**Published:** 2021-03-05

**Authors:** Xiaocong Zhang, Ziqi Chen, Jiabin Zang, Chun Yao, Jian Shi, Ruqiong Nie, Guifu Wu

**Affiliations:** 1 Department of Cardiology, Sun Yat-Sen Memorial Hospital of Sun Yat-Sen University, Guangzhou, China; 2 Department of Cardiology, The Eighth Affiliated Hospital of Sun Yat-Sen University, Shenzhen, Guangdong, China; 3 Guangdong Innovative Engineering and Technology Research Center for Assisted Circulation, Shenzhen, China; 4 NHC Key Laboratory of Assisted Circulation, Sun Yat-Sen University, Guangzhou, Guangdong, China

**Keywords:** myocardial infarction, lncRNAs, diagnostic models, support vector machine, weighted gene co-expression network analysis

## Abstract

Currently, the role of lncRNA in myocardial infarction (MI) is poorly understood. 17 co-expression modules were determined, specifically, the greenyellow, saddlebrown, grey60, royalblue, lightgreen, white, and pink modules were specifically expressed in the acute phase of MI, and brown, darkred, and royalblue, while greenyellow modules were specifically expressed in MI compared with CAD. 12 time-dependent of lncRNA/mRNA clusters with consistent expression trends were also identified. MI-associated modules were mainly enriched to immune, cell cycle, and metabolic pathways. We further obtained a network of 1816 lncRNA-mRNAs with higher expression correlations among these lncRNAs by analyzing the topological properties of the network. Herein, lncRNA RP11-847H18.2 and KLHL28, SPRTN, and EPM2AIP1 were determined as gene markers specifically expressed in MI, and they demonstrated a high predictive performance for MI diagnosis and prognosis. Three drugs, namely, Calcium citrate, Calcium Phosphate, and Calcium phosphate dihydrate, were identified as potential precursors of MI. Finally, gene and lncRNA diagnostic models were developed based on these genes and lncRNAs, with their AUCs averaged above 0.89 in both training and validation datasets. The findings of this study improve the diagnosis and prognosis of MI and personalized treatment of MI.

## INTRODUCTION

Myocardial infarction (MI) is an acute cardiovascular disease with high mortality and disability [1]. On the basis of atherosclerotic stenosis of coronary arteries, MI is an acute myocardial necrosis caused by plaque rupture and sudden obstruction of the coronary artery lumen by some stimuli, resulting in continuous and severe hypoxia-ischemia of myocardial tissues (the innervation of the infarcted vessel). After the occurrence of MI, apoptotic cascade will be activated and cardiomyocytes will become necrotic, due to persistent hypoxia and ATP deficiency in cardiomyocytes. Necrotic cardiomyocytes activate the immune system, subsequently leading to the excessive production of inflammatory response [2]. Therefore, it is important to explore the molecular mechanisms of MI pathogenesis and identify diagnostic and prognostic biomarkers and therapeutic targets.

Long noncoding RNAs (lncRNAs) are a class of RNA molecules with a transcript length of ≥200nt. LncRNAs do not encode proteins, but regulate gene expression at epigenetic, transcriptional and post-transcriptional levels through gene imprinting, chromatin reconstruction, regulation of cell cycle, variable shear, and mRNA regulation, participating in various functional processes such as cell metabolism, growth, differentiation, apoptosis and death [3, 4]. LncRNAs play important regulatory roles in the pathological processes of AMI, including in cardiomyocyte apoptosis, inflammatory response, angiogenesis, fibrosis repair and cardiac remodeling. Studies proved that some differentially expressed lncRNAs, for instance, aHIF [5], ANRIL [5], KCNQ10T1 [6], MIAT [7], ZFAS1 [8] and CDR1AS [9], have critical functions in acute myocardial infarction (AMI) and demonstrated potentials of serving as myocardial infarction-specific biomarkers or key lncRNAs in the development regulation of AMI. Moreover, Liu Cuiyun et al [10] found that lncRNA CAIF inhibits myocardial autophagy and myocardial infarction by blocking the expression of p53-regulated myocardin. These previous findings indicated that lncRNAs have key functions in AMI, but the mechanism of lncRNA regulation in myocardial infarction is far from clear. Therefore, to investigate the specific role of lncRNAs in myocardial infarction and the related regulatory mechanisms are of high significance.

In this study, 12 time-dependent lncRNA/mRNA clusters with consistent expression trends and 1816 lncRNA-mRNA networks were obtained from a co-expression network of mRNA and lncRNA established. We also determined specific expressions of lncrNARp11-847H18.2 and three genes (KLHL28, SPRTN, EPM2AIP1), which showed strong potential in MI diagnosis. Finally, a lncRNA/mRNAs-based diagnosis model was constructed with the corresponding lncRNA and mRNAs.

## RESULTS

### Identification of modules for different stages of specific expression

**Figure 1:**
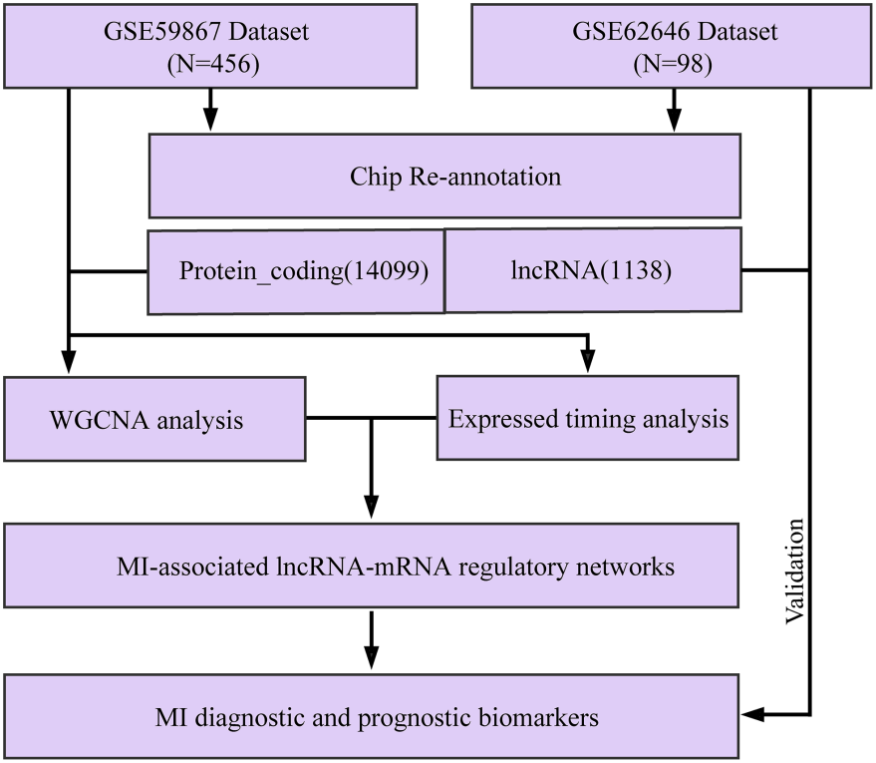
**Workflow diagram.**

**Figure 2:**
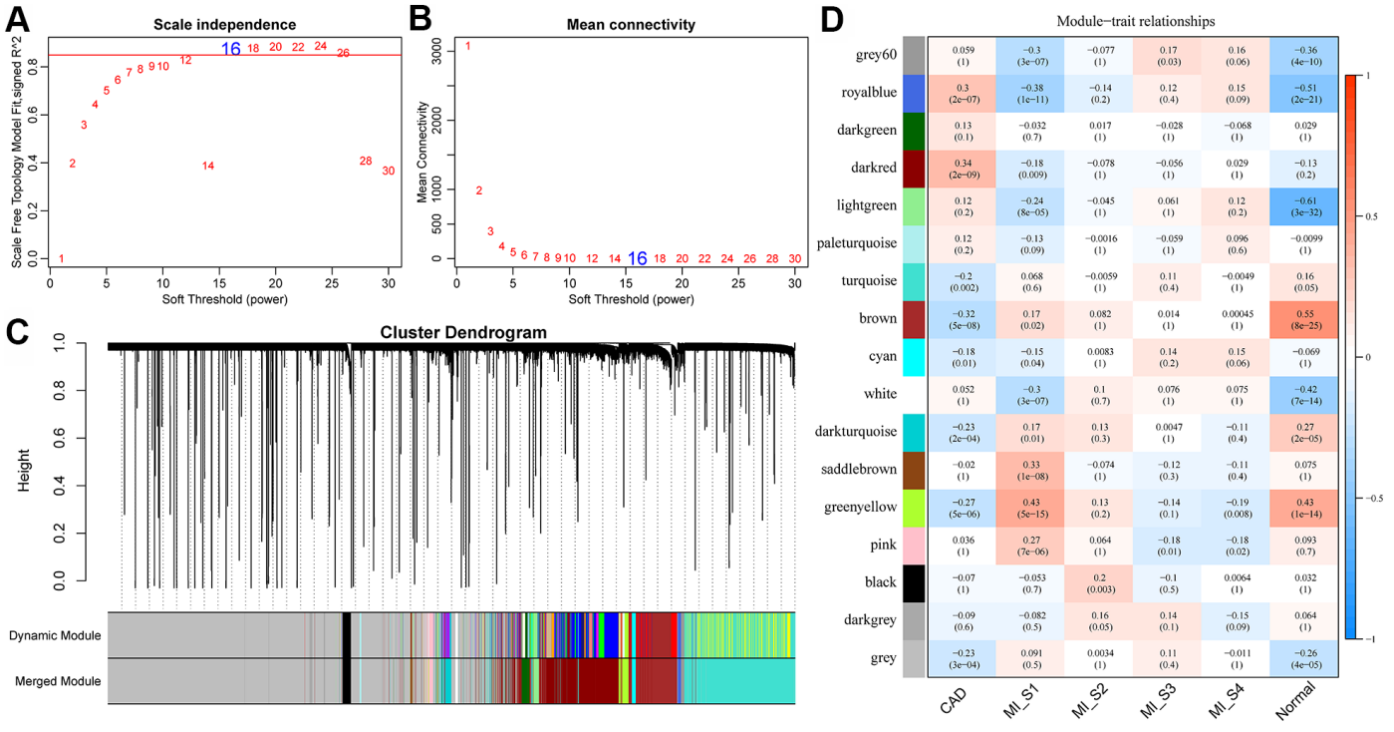
**Construction of weighted gene co-expression network and identification of disease-related modules.** (**A**, **B**) Determination of soft-thresholding power in the weighted gene co-expression network analysis (WGCNA). (**A**) Analysis of the scale-free fit index for various soft-thresholding powers (β). (**B**) Analysis of the mean connectivity for various soft-thresholding powers. (**C**) Dendrogram of all differentially expressed genes/lncRNAs clustered based on a dissimilarity measure (1-TOM). (**D**) Correlation distribution of feature vectors of each module with four time points and CAD.

The flow chart of our execution is shown in Figure 1. The "WGCNA" package in R was used to group Gene/lncRNA with similar expression patterns into modules by average-linkage hierarchical clustering. In this study, with β = 16 (scale-free *R*^^2^= 0.87) power serving as the soft threshold to ensure a scale-free network (Figure 2A, 2B). A total of 17 modules were identified (Figure 2C). The correlation between each module and feature was calculated respectively. We found that the acute phase (MI_S1) at four time points was highly correlated with multiple modules expressed in MI_S1 (*p* <1e-4), which were Greenyellow, SaddleBrown, Grey60, RoyalBlue, Lightgreen, White, and Pink (Figure 2D). However, the correlation between the other three time points and each module was weak, indicating that the greatest transcriptome changes in MI occurred at the acute phase. Compared with the four time points, CAD also showed a high correlation with several modules (*p* <1e-4), such as Brown, Darkred, Royalblue, Greenyellow, etc., In addition, we obtained data sets GSE57338 for Failing Hearts and controls from the GEO database, and calculated the relationship between eigenvectors of each module and healthy controls using the same method (Figure 2C), The results showed that the healthy control samples were highly correlated with multiple modules, similar to MI_S1, but opposite to CAD, suggesting that multiple transcriptome changes take place in the process of MI compared with the normal control group.

### Functional dimensions of MI related modules and expressed timing analysis

**Figure 3:**
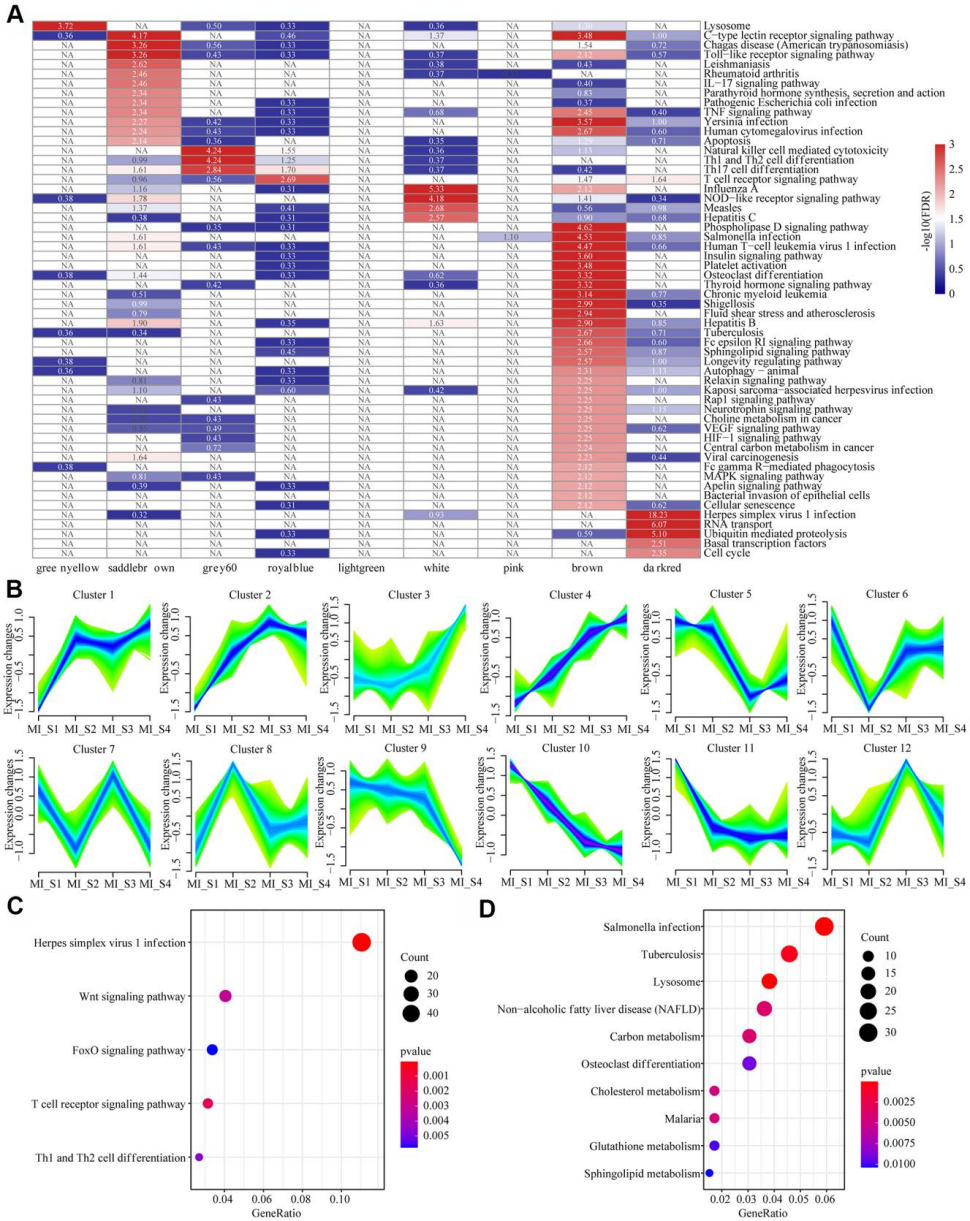
**Functional enrichment analysis of MI related modules and timing analysis of MI gene expression.** (**A**) Heatmap of 9 modules enriched to KEGG Pathway, color indicates log10 (FDR), NA indicates unenriched. (**B**) Time series analysis of 12 expression patterns of gene expression at different time points. (**C**) Sustained expression of elevated gene sets enriched to the KEGG pathway at different time points. (**D**) The KEGG pathway was enriched for a set of genes with decreased expression at different time points. The different colors indicate enrichment significance and the dot size indicates the number of enriched genes.

KEGG functional enrichment analysis was performed for a better understanding of the functional implications of the nine MI-related modules. When FDR<0.01, genes were enriched to a total of 56 modules (Figure 3A), but the pathways these modules enriched to showed less intersection, suggesting that genes in different co-expressed modules may be involved in different biological processes in time or space. We also observed that the genes in these modules were not only related to multiple viral infection pathways such as Human T-cell virus 1 infection, Pathogenic *Escherichia coli* infection, Yersinia infection, and Human Syractomiae infection, but also to Th1 and Th2 cell differentiation, Th17 cell differentiation, T cell receptor signaling pathway, IL-17 signaling pathway and some other immune pathways. In addition, these genes were enriched into cell cycle, basal transcription factors, RNA transport, cellular senescence, apoptosis and other cell proliferation and apoptosis pathways. These results suggested that MI is a complex and systematic process involving cell proliferation, apoptosis, and immunity. Considering that MI_S2\S3\S4 did not show specific relation to co-expression module, we analyzed the patterns of gene and lncRNA expressions at different time points based on time series, and determined 12 expression patterns (Figure 3B). For example, Cluster 1 showed increased expression at MI_S2 and remained at a high level since then, while Cluster 11 demonstrated an opposite pattern to that of Cluster 1. Moreover, the expression of Cluster 3 increased continuously with time, while that of Cluster10 was the opposite. The distribution statistics of genes and lncRNAs in each Cluster (Supplementary Figure 1A) showed that the proportion of lncRNAs in all the expression patterns were low, which was consistent with the background distribution characteristics dominated by PCG. These expression patterns indicated that the transcriptome was changing continuously at different time points in MI, and these changes may lead to different disease progression. For KEGG pathway enrichment analysis, we selected the gene set showing continuously increased expression (Cluster 4) and the one with continuously decreased expression (Cluster 10). We observed that Cluster 4 was mainly enriched to the Wnt Signaling pathway, FoxO signaling pathway, T cell signaling pathway, and other signaling pathways (Figure 3C), and Cluster10 was mainly enriched in Sphingolipid metabolism, glutathione metabolism, cholesterol metabolism, carbon metabolism and other metabolic pathways (Figure 3D). This suggested that the immune-related pathways of MI patients are gradually enhanced, by contrast, the carbon metabolism-related pathways are weakened over time.

### Identification of MI-associated lncRNA-mRNA regulatory networks

**Figure 4:**
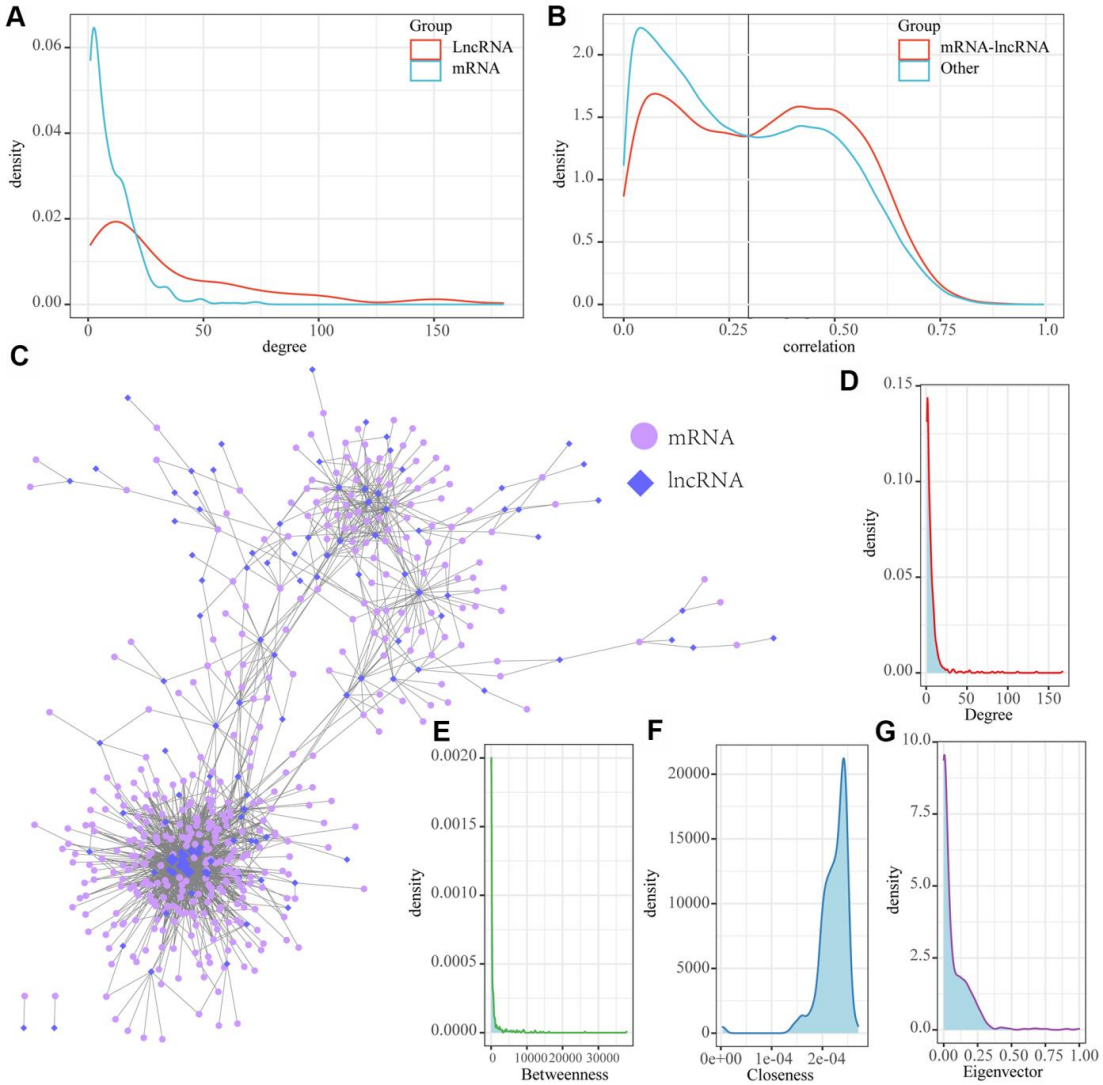
**MI-associated LncRNA-mRNA regulatory networks.** (**A**) Distribution degree of lncRNA and mRNA nodes in lncRNA-mRNA interaction networks; (**B**) Distribution of lncRNA and mRNA correlations in LncRNA-mRNA regulatory networks and lncRNA-mRNA correlations in non-lncRNA-mRNA networks; (**C**) MI-associated lncRNA-mRNA regulatory networks. (**D**) Distribution degree of the network. (**E**) Median centrality distribution of the network. (**F**) The near-central distribution of the network. (**G**) The eigenvector centrality distribution of the network.

We examined the distribution of genes and lncRNAs in MI-associated modules and two continuously changing expression patterns, and observed a significantly low proportion of lncRNAs in seven of these modules (FDR < 0.05), suggesting that lncRNAs may be indirectly involved in MI development through multiple regulatory pathways. Based on this, a new computational method was developed to identify the lncRNA-mRNA interaction network in MI. This was achieved by integrating paired expression profiles of genes/lncRNAs from disease-related co-expression modules into gene expression datasets according to the regulatory interactions among mRNAs, lncRNAs and miRNAs. Here we determined a network of 5320 lncRNA-mRNA interactions incorporating a total of 510 mRNAs and 154 lncRNAs (Table 1). The mRNAs and lncRNAs in the network showed significantly different degrees of distributions, with the average degree of lncRNAs greater than that of mRNA, suggesting that lncRNAs are more likely to be the hub nodes in the network (Figure 4A). The distribution of mRNA-lncRNA correlations was further analyzed, and the results demonstrated that there was a significant correlation between the distribution of between lncRNAs and mRNAs (Figure 4B). This suggested that the association between lncRNAs and mRNAs may have stronger interactions in MI than other gene correlations. Therefore, the intersection of different mRNA-lncRNA correlations were acted as the threshold, we further identified lncRNAs/mRNAs with correlations higher than 0.3 in the interaction network as MI-associated lncRNA-mRNAs, resulting in a network of 1816 lncRNA-mRNA interactions incorporating 417 genes and 112 lncRNAs (Figure 4C). Further analysis detected several nodes with the highest and lowest degrees of distribution consistent with the biological network characteristics (Figure 4D). In addition, the network showed a median distribution (Figure 4E), the same near-centrality distribution (Figure 4F), and eigenvector centrality distribution (Figure 4G). All these results indicated that MI-associated lncRNA-mRNA is a canonical biological regulatory network centered on lncRNAs.

**Table 1:** Genes and lncRNAs in MI-related modules or expression patterns.

**Tag**	**LNC**	**PCG**	**p.value**	**FDR**
brown	31	1211	7.40E-16	6.66E-15
darkred	57	1974	2.69E-22	2.69E-21
greenyellow	5	248	2.31E-05	0.000162
grey60	7	104	0.268219	0.536437
lightgreen	4	145	0.003388	0.016941
pink	4	154	0.00197	0.011819
royalblue	0	76	NA	NA
saddlebrown	4	41	0.563794	0.563794
white	1	56	0.011879	0.047516
Cluster4	86	1264	0.04585	0.13755
Cluster10	46	1170	1.02E-08	8.14E-08

### Functional enrichment analysis of MI-associated lncRNAs-mRNAs network

**Figure 5:**
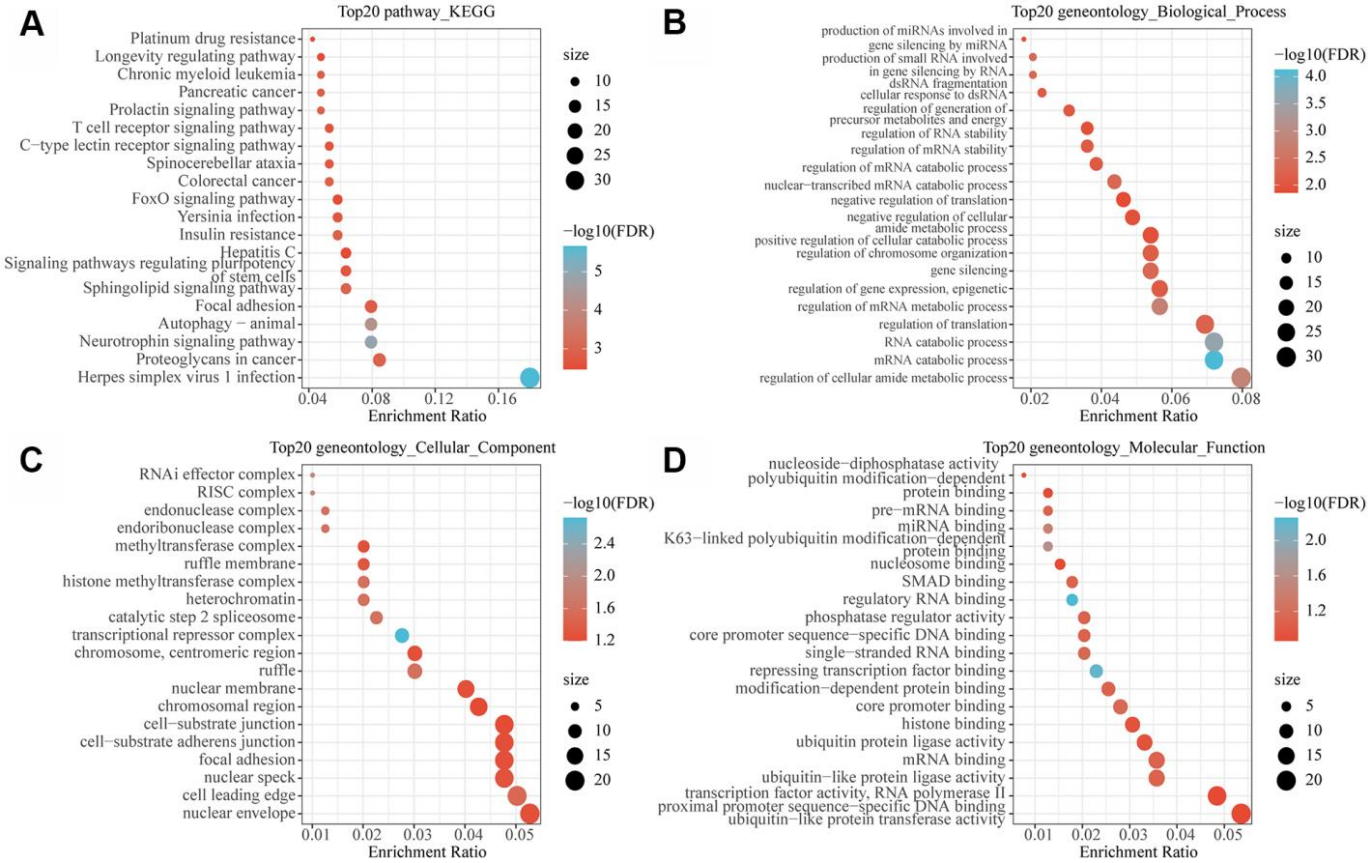
**Functional enrichment analysis of MI-related lncRNA-mRNA networks.** (**A**) The 20 most significant KEGG pathways enriched by MI-related lncRNA-mRNA network. (**B**) The 20 most significant GO biological processes enriched by MI related lncRNA-mRNA network. (**C**) The 20 most significant GO biological processes enriched by the MI-related lncRNA-mRNA network. (**D**) The 20 most significant GO molecular functions enriched by the MI-related lncRNA-mRNA network. Node size in the figure represents the number of genes enriched in the pathway, and color represents the significance enriched.

To observe the function of the MI-associated lncRNA-mRNA network, the genes in the network were subjected to functional enrichment analysis of GO and KEGG pathway. The analytical data demonstrated that these genes were mainly enriched to autophagy, insulin resistance and some other pathways (Figure 5A). Previous studies found that autophagy in blood cardiomyocytes can provide the energy necessary for cell survival by removing disordered organelles or senescent proteins [11]. For example, NR4A2 knockdown exacerbates cardiomyocyte apoptosis, and upregulation of NR4A2 is regarded as an adaptive response to ischemia-induced cardiomyocyte apoptosis [12]. Insulin resistance is considered as a key risk factor for adverse metabolic and cardiovascular diseases [13]. In the heart, insulin receptor substrates (IRS) are key regulators of the insulin signaling pathway. Under the activation of insulin receptor (INSR), IRS promotes glucose transporter protein 4 (SLC2A4, also known as GLUT4) translocation and initiates cell survival pathways in cardiomyocytes [14], in addition, reduced insulin sensitivity in ischemic myocardium contributes to defective IRS1 function [15, 16]. These findings demonstrated that the lncRNA-mRNA network may be involved in the development of MI through regulating autophagy and insulin resistance. Moreover, these genes were also found to be enriched in a variety of metabolic and decomposition processes (Figure 5B). The metabolic process of degrading damaged proteins or organelles is the main mechanism of autophagy. The enrichment results of cell components showed that these genes were enriched into a variety of complexes related to transcriptional regulation and histone modification (Figure 5C). In addition, molecular functions were mainly enriched in multiple RNA binding, nucleosome binding and modification−dependent protein binding (Figure 5D), suggesting that these genes are also involved in chromatin modification and regulation. Epigenetic mechanism plays a key role in the regulation of mammalian gene expression. Differential histone modification represents a typical epigenetic mechanism, meaning that epigenetic modification also participates in the occurrence and development of MI.

### MI diagnostic and prognostic biomarkers were identified by lncRNA-mRNA network mining

**Figure 6:**
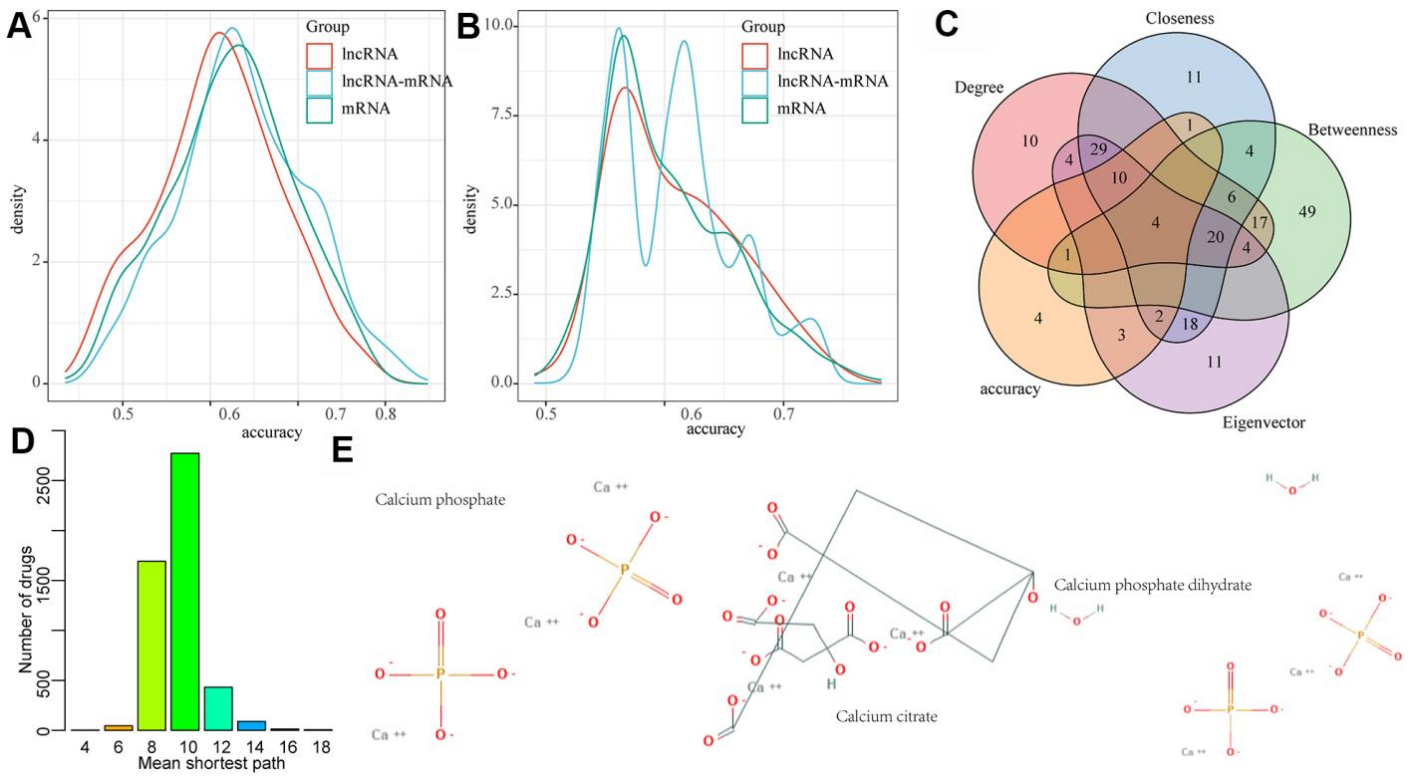
**LncRNA-mRNA network mining was used to identify MI diagnostic and prognostic biomarkers.** (**A**) The distribution of diagnostic accuracy of each lncRNA, mRNA, lncRNA-mRNA pair in the network for MI; (**B**) The distribution of prognostic of each lncRNA, mRNA, lncRNA-mRNA pair in the network for MI; (**C**) The intersection of candidate RNA molecules and nodes with network degree, medium centrality, near centrality and eigenvector centrality of top 10% in the network; (**D**) Mean shortest path distribution of drugs to MI gene markers. (**E**) 2D structures of three potential MI drug molecules.

In order to examine the diagnostic performance of the lncRNAs and mRNAs in the network, linear discriminant analysis was applied to classify and predict each lncRNA, mRNA, and lncRNA-mRNA pair in the network, respectively, and their prediction accuracy distribution was calculated accordingly (Figure 6A). We observed that lncRNA, mRNA and lncRNA-mRNA presented similar classification accuracy, with an average accuracy of more than 0.6, which verified the diagnostic performance of the nodes in the lncRNA-mRNA network. In addition, we also investigated whether early changes in gene expression can also predict disease prognosis and distinguish patients with HF after MI from those without HF. Here, the classification/prediction accuracy distribution of each lncRNA and mRNA as well as lncRNA-mRNA pair in the network were evaluated in the same way (Figure 6B), and their average prediction accuracy was found to be greater than 0.55. Noticeably, the prediction accuracy of lncRNA-mRNA was significantly higher than that of lncRNA and mRNA alone. We also observed that the classification accuracy distribution of lncRNA-mRNA presented four peaks, indicating the existence of a variety of different lncRNA-mRNA combinations. Therefore, lncRNA-mRNA combinations in the peak with the highest accuracy were selected as the final candidate markers. Based on this, lncRNA-mRNA pairs with a diagnostic and prognostic accuracy greater than 0.7 were selected as candidate markers. In addition, we also found that nodes with higher network degree, intermediate number centrality, near centrality and feature vector centrality in biological networks were more likely to be the core nodes in the network regulation process. Thus, the nodes with the top 10% of network degree, medium centrality, near centrality, and eigenvector centrality were determined as the hub nodes of the network. The intersection of them and candidate markers contained a total of one lncRNA (RP11-847H18.2) and three genes (KLHL28, SPRTN, and EPM2AIP1) (Figure 6C). These four molecules combined showed a high classification performance with high network importance, and can therefore be used as diagnostic and prognostic markers of MI. In addition, we also analyzed the relationship between the three genes and drugs, obtained the protein interaction network composed of drug target genes and our three genes through STRING database, constructed the drug-target gene-disease-specific gene interaction network, and determined the shortest distribution path of drugs to these three disease-specific genes in the network (Figure 6D). We observed that the average shortest path of most drugs was 10, while that of three drugs was only 4, suggesting that these three drugs may be effective for MI treatment (Figure 6E).

### Construction of the MI diagnostic model and testing of the model

**Figure 7:**
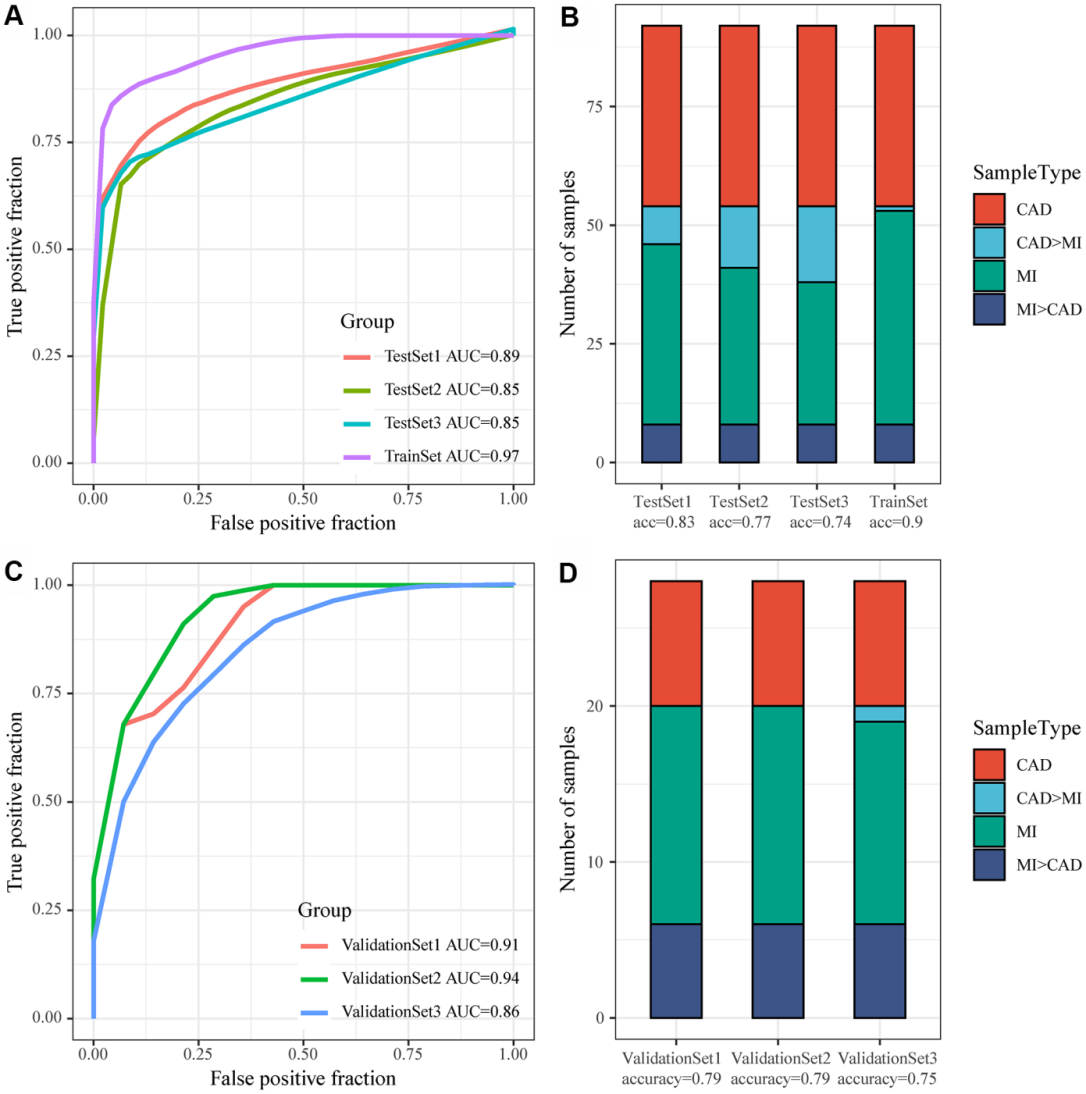
**Construction of the MI diagnostic model and testing of the model.** (**A**) ROC curve of the diagnostic model for classification of samples in the training dataset and internal validation dataset; (**B**) Classification accuracy of the diagnostic model on samples in the training set and in the internal validation set; (**C**) ROC curve of the diagnostic model for classification of samples in the validation dataset; (**D**) Classification accuracy of the diagnostic model on samples in the training set and in the validation dataset.

GSE59867 was considered as a Train dataset, to balance the proportion of control and disease groups, we randomly selected 46 samples from 73 patients in MI_S1 from the control group to form a cohort with 46 samples as a training set (TrainSet), and another 46 samples were selected from MI_S2, MI_S3, and MI_S4 to form a cohort with the control group, respectively. The three datasets used as internal validation sets were TestSet1, TestSet2, and TestSet3. Similarly, GSE62646 served as an external validation set, and equal proportions of MI samples corresponding to the three follow-up time points in the GSE62646 dataset and 14 control samples were gathered together to form three validation datasets, namely, ValidationSet1, ValidationSet2, and ValidationSet3. In the training set, one lncRNA and three genes served as features, and their expression profiles were obtained to construct a support vector machine (SVM) classification model, which was tested by a tenfold cross-validation method. The AUC of the training set was 0.97 with 90% classification accuracy, 0.89 with 83% classification accuracy in TestSet1, 0.85 with 77% classification accuracy in TestSet2, and 0.85 with 74% classification accuracy in TestSet3 (Figure 7A, 7B). We further applied the model to the external validation set GSE62646, and obtained an AUC of 0.91 and classification accuracy of 79% in ValidationSet1, an AUC of 0.94 and classification accuracy of 79% in ValidationSet2, and an AUC of 0.86 and classification accuracy of 75% in ValidationSet3 (Figure 7C, 7D). These results indicated that the diagnostic and predictive model constructed in this study can effectively distinguish MI patients from CAD controls, and that the one lncRNA and three genes can be used as reliable biomarkers for MI diagnosis.

## DISCUSSION

The aim of this study was to identify potential diagnostic and prognostic biomarkers from the GEO database for myocardial infarction (MI). In this study, a candidate lncRNA and three mRNAs as potential biomarkers of MI after performing an exhaustive literature review. Our linear discriminant analysis showed that the lncRNA-mRNA pair exhibited a better performance in predicting prognosis than using individual an lncRNA or mRNA. Our target prediction and pathway analysis found that the lncRNA-mRNA network typically regulated autophagy and insulin resistance pathways. The results of drug-target gene interaction data proved that three drugs (Calcium phosphate, Calcium citrate and Calcium phosphate dihydrate) had strong potentials in protecting patients against MI. Furthermore, we selected the largest molecular weight drug (DB11093: Calcium citrate) and evaluated the relationship with the three core genes in the network (KLHL28, SPRTN, EPM2AIP1) by molecular docking (Supplementary Figure 1B), the docking results with SPRTN, KLHL28 and EPM2AIP1L showed that the docking scores of the ligand with three receptor proteins were -5.4 kcal/mol, -5.8 kcal/mol and -5.0 kcal/mol, respectively. The docking scores of this ligand with the receptor all reached 5.0 kcal/mol at a small molecular weight loudness, indicating that the molecule has some binding affinity to the three proteins. In addition, DB11093 binds tightly to the zinc ion in the SPRTN protein in the form of a three-foot chelate. The compound can also be found to interact with VAL381, ALA427 and PHE324 in the protein by hydrogen bonding when DB11093 binds to KLHL28 protein. And when DB11093 binds to EPM2AIP1L protein, it can also form hydrogen bonding interactions with ARG343, ASN334 and LYS309 with supporting effects. The above results suggested that DB11093 has some affinity with SPRTN, KLHL28 and EPM2AIP1L and may become an inhibitor of the three targets. To the best of our knowledge, this is the first study that discovers a unique lncRNA-mRNA signature for MI detection and its pathogenic mechanism.

Our lncRNA-mRNA displayed excellent performance in MI diagnosis and prognosis. SPRTN gene has recently been found to be functional in translating DNA synthesis and preventing mutations [17, 18]. *In vivo* and *in vitro* characterization of identified mutations revealed that SPRTN plays an important role in the prevention of DNA replication stress during general DNA replication and in the regulation of replication-associated G2/M-checkpoints [19]. Mutations of SPRTN could lead to the early onset of hepatocellular carcinoma and genomic instability [19]. Moreover, the absence of EPM2AIP1 in mice impairs the allosteric activation of GS by glucose 6-phosphate, reduces liver glycogen synthesis, increases liver fat, and promotes liver insulin resistance [20]. LncRNA RP11-847H18.2 and mRNA KLHL28 has not been previously reported. Compared with using lncRNA and mRNA alone in diagnosing and predicting MI prognosis, the lncRNA-mRNA pair developed in this study showed a higher discriminatory power, demonstrating a highly effectiveness of serving as a diagnostic and prognostic tool during MI detection.

Our functional enrichment analysis study revealed that the lncRNA-mRNA network is associated with autophagy and insulin resistance. Previous reports detected a sharp increase in autophagy during the reperfusion phase of cardiac ischemia [21, 22]. Kanamori et al. also confirmed that autophagy activity increases in the subacute and chronic phases of heart ischemia in MI mouse models [23, 24]. Noticeably, autophagy activity plays an even more critical role in the margin of infarction than in remote areas of the myocardium [25]. Insulin resistance is considered to be an adverse metabolic and a key risk factor for developing cardiovascular diseases [13], and insulin receptor substrates (IRS) are key modulators of insulin signal transduction pathways in the heart. After the activation of insulin receptor (INSR), IRS will promote the translocation of glucose transporter 4 (SLC2A4, also known as GLUT4) and initiates the cell survival pathway of cardiomyocytes [14]. The reduced insulin sensitivity in ischemic myocardium contributes to defective IRS1 function [15, 16]. These results indicate that the lncRNA and mRNAs in the network developed by this study may be involved in the development of MI through regulating autophagy and insulin resistance.

There are still some limitations in this study. For example, our sample size was relatively small for the evaluation of the correlation between the lncRNA-mRNA pair and the severity of MI or long-term clinical results. Thus, further basic research is required to verify the accuracy and clinical applicability of the lncRNA-mRNA pair in MI detection.

## CONCLUSIONS

In conclusion, this study was the first to identify a unique gene signature (lncRNA RP11-847H18.2, mRNA KLHL28, SPRTN and EPM2AIP1), which allows an early detection of human MI. The gene signature specifically targets autophagy and insulin resistance, and may be involved in pathologies of MI.

## MATERIALS AND METHODS

The experiment was conducted briefly as follows: data collection, co-expression module identification, time series analysis, enrichment analysis, feature selection, followed by classifier construction and validation.

### RNA expression spectrum

GSE59867 expression profile data set of MI patients was acquired from the Gene Expression Omnibus (GEO) database (http://www.ncbi.nlm.nih.gov/geo/) [26] on the platform of Affymetrix Human Gene 1.0 ST Array. The dataset consisted of 456 samples, including 111 PATIENTS with ST-segment elevation MI and 46 patients with stable CAD without previous history of MI. The corresponding mRNA expressions were obtained from peripheral blood mononuclear cells at four time points, specifically on the day of admission (day 1, MI MI_S1), at discharge (4 to 6 days after MI, MI_S2), 1 months after MI (MI_S3) and 6 months after MI (MI_S4). The expression profiles of 73 out of 111 patients were detected at the four time points, so their expression profiles and 46 CAD samples served as controls in this study. In addition, we also downloaded the expression spectrum from the same platform dataset GSE62646 [27]. In this dataset, a total of 98 samples contained 28 patients with ST-segment elevation MI and 14 patients with stable CAD without MI history. Corresponding expression profiles were obtained from peripheral blood mononuclear cells at the day of admission (day 1 of MI, MI_S1), at discharge (4-6 days after MI, MI_S2) and 6 months after MI (MI_S4).

Probe sequences of GSE59867 and GSE62646 datasets were first aligned to the genome (GRCH38.p13) by chip reannotation to obtain probe mapping transcript IDs, and each transcript cluster was assigned to the Ensembl gene ID. Then, for transcription clusters with Ensembl gene ID, "LincRNA", "sense_intronic", "sense_overlapping", "antisense", "processed_transcript", "3 prime_overlapping_ncrna", "antisense_RNA", "TEC", and "bidirectional_promoter_lncRNA" were retained, but only "non_coding" cluster was considered as a lncRNA [28]. Finally, 1138 lncRNAs were collected after the removal of redundant transcripts. In addition, the cluster with annotation type "protein_coding" was retained, and was considered as coding genes, here, we obtained a total of 14,099 coding genes.

The original data of GSE59867 and GSE62646 were processed by R Software package AFFy [29], and the expression matrix of probe was obtained by using RMA standardization. The probe was mapped to the gene, and the median value was taken as the expression value of the gene when multiple probes were mapped to the same gene. In this way, the expression matrixes of genes and lncRNAs were finally set up.

### Expression analysis of lncRNAs and mRNAs, and the construction of weighted co-expression network

To better identify disease-related genes and lncRNAs, the expression profiles of lncRNAs and genes were combined for establishing a weighted co-expression module. Specifically, RNA expression data profiles of genes and lncRNAs were first tested to verify whether the samples, genes, and lncRNAs were qualified. Then, weighted gene co-expression network analysis (WGCNA) [30] package in R was applied to construct a scale-free co-expression network for the genes and lncRNAs, and the Pearson's correlation matrices and average linkage method were both performed for pair-wise testing. Then, a weighted adjacency matrix was constructed using a power function A_mn_ = |C_mn_*|*^β^ (C_mn_ = Pearson's correlation between gene/lncRNA m and gene/lncRNA n; A_mn_ = adjacency between gene/lncRNA m and gene/lncRNA). β served as a soft-thresholding parameter that emphasizes strong correlations between gene, lncRNAs and removes weak correlations. Topological overlap matrix (TOM), which measures the connectivity of a gene/lncRNA to the network, is defined by the sum of a gene adjacency with all other gene/lncRNAs for network gene/lncRNA ration. After deciding the power of β, the adjacency was converted into a TOM, and the corresponding dissimilarity (1-TOM) was calculated. Gene and lncRNAs with similar expression profiles were accordingly classified into corresponding modules, and average-linkage hierarchical clustering was conducted according to the TOM-based dissimilarity measure with a minimum size (gene/lncRNA group) of 30 for the genes/lncRNAs dendrograms. To further analyze the module, we calculated the dissimilarity of module Eigen of genes/lncRNAs, decided a cut line for module dendrogram and some modules were merged.

### Identification of disease-related co-expression modules and lncRNA/miRNA clusters with consistent expression trends during MI progression

Co-expression modules eigengenes (MEs) were considered as the main components in the principal component analysis of each module, and the expression patterns of all the genes and lncRNAs can be generalized into an expression profile with a single characteristic RNA in a given module. Therefore, correlations between ME and MI features at different time points were computed to identify MI-related co-expression modules. The R package "Mfuzz [31] was used to detect lncRNA and miRNA clusters with consistent expression trends during MI progression.

### Functional enrichment analyses

Gene Ontology (GO) and Kyoto Encyclopedia of Genes and Genomes (KEGG) pathway enrichment analysis was performed using the R package clusterProfiler [32] for genes to identify over-represented GO terms in three categories (biological processes, molecular function and cellular component) and KEGG pathway. For both analyses, *p* < 0.05 was considered to denote a statistical significance.

### Regulatory interactions between miRNA-mRNAs and miRNA-lncRNAs

A total of 416312 non-redundant miRNA-mRNA interactions were obtained by acquiring all the regulatory relationships of miRNA-mRNA from the miRanda [33], miRTarBase [34], TargetScan [35], and starBase [36] databases. By predicting the miRNA-lncRNA interactions on the starBase [36] and miRcode [37] databases, 295,601 non-redundant miRNA-lncRNA relationships were retained.

### Identification of MI-associated lncRNA-mRNA regulatory networks

Based on the ceRNA hypothesis [38, 39], a candidate lncRNA or mRNA is determined if it satisfies all of the following conditions: (1) the miRNA shared by mRNA and lncRNA is significantly enriched (FDR<0.01 as determined by hypergeometric test); (2) the mRNA-lncRNA is significantly enriched in the same disease-associated co-expression module or in the same expression pattern (Cytoscope for visualization) [40]. Next, the topological properties of the network and the distribution of lncRNA-mRNA correlations in the network were analyzed to select lncRNA-mRNA pairs with correlations greater than 0.3 as candidates for MI correlation examination.

### Network construction of gene markers and drug targets

To investigate the potential drug effects of these RNA markers, we identified a total of 16,196 drug-gene interactions from the DrugBank V5.1.7 database [41]. These drug target genes and RNA marker genes were co-mapped to the String V11.0 database (https://string-db.org/) [3] to obtain gene interaction information for the construction of a drug-gene-MI marker network. The shortest path from each drug to MI marker gene in the network was calculated, and the average shortest path of drug to MI marker was determined as the treatment candidate.

### Construction of MI diagnostic prediction model and evaluation of model prediction performance

As a supervised learning model of machine learning algorithms, support vector machine (SVM) analyzes data and identifies patterns. A support vector mechanism creates a hyperplane for classification and regression in high or infinite dimensional space. MI gene markers and lncRNA markers were used to construct a diagnostic prediction model based on SVM [42] classification for the prediction on the MI and CAD samples. Given a set of training samples and each tag belongs to two categories, a SVM training algorithm establishes a model and assigns new instances to one category or another to allow a non-probabilistic binary linear classification. GSE59867 was taken as Train dataset, in order to balance the proportion between the control group and the disease group, we randomly selected 46 samples from 73 patients in the MI_S1 and 46 samples from the control group to form a queue as training set TrainSet. 46 samples were extracted from MI_S2, MI_S3 and MI_S4 and formed three data sets (TestSet1, TestSet2 and TestSet3, respectively) as the internal validation sets for the control group. Similarly, GSE62646 was regarded as the external validation set, and the three validation datasets (ValidationSet1, ValidationSet2 and ValidationSet3, respectively) were composed of the samples with the same proportion as the control group. Specifically, there were 14 control samples from the MI samples corresponding to the three follow-up time points in the GSE62646 dataset. The model was constructed in the training dataset, and its classification performance was examined by using the ten-fold cross-validation method. The established model was then used to predict the samples in the validated dataset. The predictive capability, predictive sensitivity and specificity of the model to MI were analyzed and evaluated by the area under the ROC curve (AUC).

## Supplementary Material

Supplementary Figure 1
