# Supplementary material for: LncRNA-mRNA co-expression analysis discovered the diagnostic and prognostic biomarkers and potential therapeutic agents for myocardial infarction
Source: Aging (Albany NY). 2021 Mar 5;13(6):8944–59. doi: 10.18632/aging.202713 (PMC8034908; doi:10.18632/aging.202713)
Supplement: Supplementary Figure 1 [file aging-13-202713-s001.pdf]

SUPPLEMENTARY FIGURE

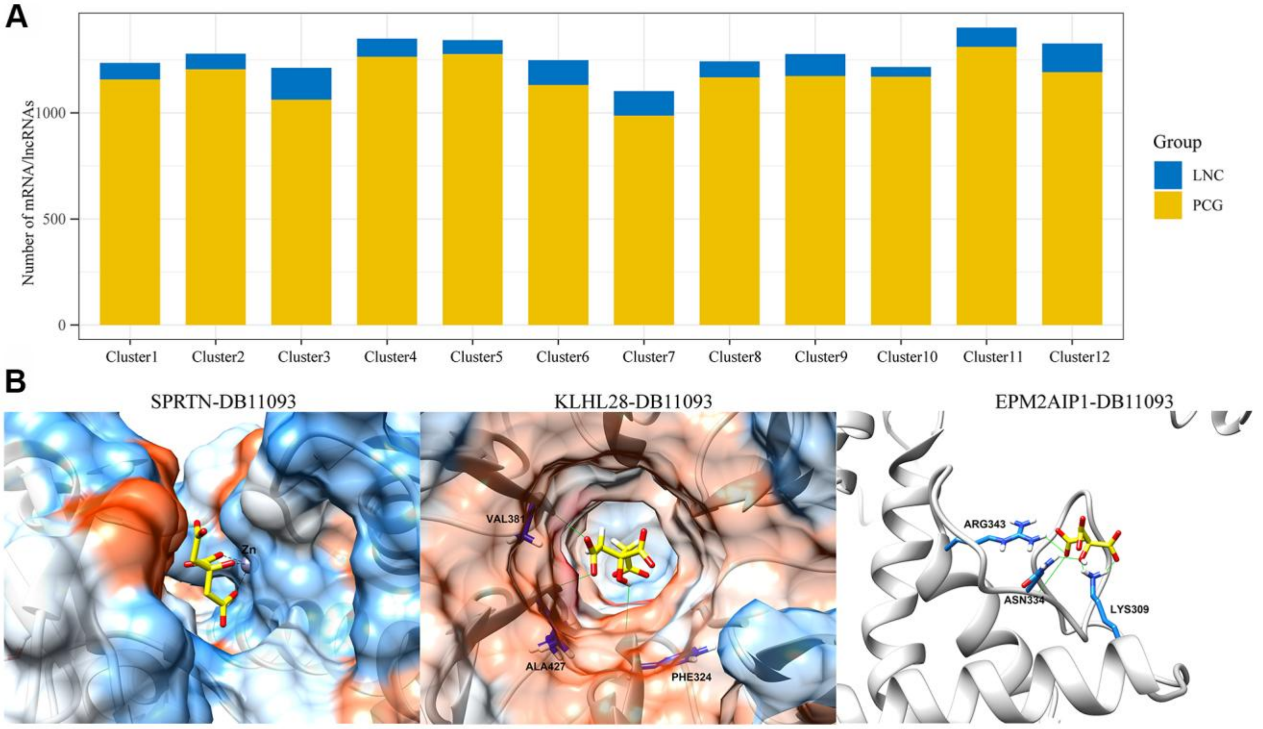

Supplementary Figure 1. (**A**) Distribution statistics of genes and lncRNAs under various co-expression patterns. (**B**) Diagram of interaction between KLHL28, SPRTN and EPM2AIP1 and the drug DB11093, Coordination bonds are shown as the black dotted line and hydrogen bonds as the green solid line. DB11093 is shown as a yellow stick and the color of the heteroatoms on it is shown as an element. In addition, hydrophobic surfaces were added in images (**A**, **B**) to better observe the binding sites of the ligand proteins.
